# The Book World of Medicine and Science

**Published:** 1905-03-04

**Authors:** 


### The Book World of Medicine and Science.

Human Embryology and Morphology. By Arthur Keith. (London: Edward Arnold. Price 21s. 6d. net.)

During the last few years so great has been the advance in our knowledge of the subject, that this edition of Keith's "Embryology" is practically a new work by the same author, and it will be welcomed by all who are interested in the study of the morphology and embryology of the human species. Unlike the majority of text-books on this subject the volume before us is pleasant to read, and the descriptions are easy to follow. The text is copiously illustrated. The figures and illustrations, most of which are original, are excellent, and their interpretation is rendered much easier by their explanatory labels being placed alongside, instead of the old method being followed of complicated references to a footnote. The old device of "pointing a moral to adorn a tale" is justified and made use of by Mr. Keith in his many references to anatomical and surgical conundrums, which he explains on embryological and morphological grounds. These references not only breathe life into this somewhat dry, and certainly very difficult, subject, but render the book interesting alike to the surgeon, physician, and pure anatomist. Mr. Keith points out for instance how rare amongst mammals is the occurrence of inguinal and femoral hernia; so rare that it may be considered almost a human peculiarity. The condition results from the adoption of the upright mode of progression, and is due to the unique form of Poupart's Ligament in man, it being scarcely developed in any other animal; the decreased size of the conjoined tendon in man; the heaviness of the upright body requiring a wide pelvis; a more direct inguinal canal; and, perhaps more important than all, the compression of the abdominal contents by the contraction of the musculature of the abdominal parietes. Mr. Keith's description of the descent of the testicle is a particularly happy one; he does not consider that the gubernaculum performs its function by contracting and drawing down the testis, but rather it is "an invading army of cells," invading the abdominal wall and dragging with it the peritoneum in the iliac fossa, on which the testis is dragged like "a log on a sledge." Hitherto the appendix has always been relegated into the list of vestigial remains, but it is pointed out that although the anthropoids, lemurs and opossums have a corresponding organ, a distinctly marked appendix is only seen in man. It is a highly-developed "cœcal apex," and although it may be dispensed with, it is no more a vestigial remnant than the tonsil. The third month seems to be a critical time in the history of the evolution of the human embryo. At this time the segments of the os innominatum should grow together and form the acetabulum; if the development is arrested at this period, congenital dislocation of the now fully-formed femoral head results. Likewise cleft palate and imperforate anus may result from arrested growth at this, the reptilian stage.

The British Journal of Children's Diseases. Vol. I. Edited by George Carpenter, M.D. (London; Adlard and Son. 1904. Price 15s. net.)

No better justification could be desired for adding yet one more medical periodical to the already long list than the success attained by this journal in the one year of its existence. In our review of the first number, which appeared in January of last year, we pointed out that in respect to children's diseases there was a distinct gap in British medical journalism, and we anticipated that, if the high standard of the first number were maintained, those who had initiated the enterprise might rest assured of success. This anticipation has been amply fulfilled, and we may safely assert that every one who practises medicine will find in the pages of this journal new facts and suggestions which will much more than repay the time devoted to their perusal. Dr. Carpenter and those who have assisted him may congratulate themselves on the result of their endeavours, and on having earned the gratitude of the medical profession.

Cancer and its Treatment. By A. W. Mayo Robson, D.Sc., F.R.C.S. (London: Baillière, Tindall, and Cox. Pp. 69. Price 3s. 6d.)

Mr. Robson's Bradshaw Lecture for 1904 is a sermon preached from the text "early operation is not merely palliative but curative." It is therefore specially acceptable after the pessimism of some surgeons, a pessimism which has affected a considerable number of the general public rendering them unwilling to accept operation and so lessening the chances of cure. A precancerous condition is recognised, and throughout the lecture it is pointed out, time after time, that this is the ideal stage for operation. Figures collected from a large number of sources are given substantiating all the statements in the book and forming in themselves most interesting reading.

The Infectivity and Management of Scarlet Fever. By T. W. Gordon Pugh, M.D., B.S.Lond. (London: J. and A. Churchill. Pp. 21. Price 1s.)

In this paper, issued by the Medical Officers of Schools Association, Dr. Pugh discusses the various modes of infection and gives details of home and hospital treatment, pointing out some of the drawbacks of the latter. Though short, the paper gives many interesting facts and figures, and is well worth perusal.

The Teachings of Zoroaster. By S. A. Kapadia. (London: John Murray. 1905 Pp. 104. Price 2s. net.)

Dr. Kapadia has accomplished the difficult task of combining the strictest brevity with an easy and smooth style, and he has contrived to produce a book which will afford a couple of very pleasant hours to anyone of a contemplative mind. Our only regret is that the author has not found space to say a little more about the hygienic features of the religion of the Parsis.

Street's Newspaper Directory, 1905 (London: G. Street and Co., Limited, 8 Serle Street, W.C. Price 3s. 6d.)

This Directory contains very complete lists of the newspapers and periodicals published throughout the United Kingdom, the Colonies, and India, with various useful details concerning each publication. There are also some excellent maps of England, Wales, and Scotland, showing at a glance the number of newspapers published in the principal towns in these countries. The book is well arranged for easy reference and should prove of value to all who require such information as it contains.

Force in Advertising. (London: S. H. Benson, 1 Tudor Street, E.C.)

A well-produced pamphlet containing much sound common sense on the subject of advertising, expressed in a direct and forceful manner, with illustrations of the various methods employed by leading advertisers.

### BOOKS RECEIVED.

G. Street and Co., Limited.

"Street's Newspaper Directory, 1905."

Adlard and Son.

"The British Journal of Children's Diseases." Vol. I.

